# Drug-resistant TB treatment outcomes and factors associated with discontinuation and LTFU in Germany: No1Lost study protocol

**DOI:** 10.5588/ijtldopen.25.0543

**Published:** 2026-01-09

**Authors:** M. Gödecke, F. Eberhardt, G. Barten-Neiner, M. Knipper, N. Aepfelbach, B. Häcker, T. Bauer, M.I. Gröschel, R. Otto-Knapp

**Affiliations:** 1 German Central Committee against Tuberculosis (DZK), Berlin, Germany;; 2 CAPNETZ STIFTUNG, Hannover, Germany;; 3 Institute of the History, Theory and Ethics of Medicine, University Justus Liebig Giessen, Giessen, Germany;; 4 Helios Clinic Emil von Behring, Department of Pneumology, Chest Hospital Heckeshorn, Berlin, Germany;; 5 Department of Infectious Diseases, Respiratory and Critical Care Medicine, Charité-Universitätsmedizin Berlin, Berlin, Germany.

**Keywords:** tuberculosis, MDR/RR-TB, risk factor, social determinants, sociomedical, prospective cohort, loss to follow-up

## Abstract

Treatment discontinuation, including loss to follow-up (LTFU), is an important challenge for TB treatment, particularly for multidrug- or rifampicin-resistant TB (MDR/RR-TB). Evidence from low-incidence countries evaluating risk factors for unsuccessful outcomes is scarce. Our study aims to examine treatment outcomes and factors associated with treatment discontinuation for MDR/RR-TB patients in the German health care setting. This observational prospective, multi-centre cohort study will enrol 150 MDR/RR-TB patients in treatment centres between 2025 and 2027. Primary outcome is the description of treatment results. Exploratory analyses will be performed, including logistic regression to assess demographic, clinical, and important factors like social determinants possibly associated with treatment discontinuation. Secondary outcomes will include the descriptive analysis of outcomes and safety of the recently recommended 6–9 months and longer MDR/RR-TB regimens, barriers for adherence to TB guidelines and the clinical structures for MDR/RR-TB management. The prospective No1Lost MDR/RR-TB cohort study will improve the understanding of risk factors and barriers for treatment discontinuation and LTFU in a low-incidence setting to address high rates of treatment discontinuation in this vulnerable population with targeted interventions.

Germany is a low-incidence country for TB with a high-resource health care system. Nevertheless, treatment success for TB remains below WHO targets with 63% in 2022.^1,2^ Inconclusive treatment results due to patients transferred to unknown destinations (5.1%) or treatment discontinuation for other reasons (3.2%) are major drivers for unsuccessful TB treatment results.^3^ Early TB treatment discontinuation increases the risk of treatment failure, antibiotic resistance, and transmission of resistant TB strains.^4^ Factors associated with TB treatment discontinuation include older age, alcohol and drug use, poor health literacy, drug adverse events, treatment length, and lack of social support, especially in contexts marked by socio-economic, legal, and structural challenges, such as homelessness and uncertain immigration status.^5–10^ However, published data mainly originate from countries with high TB incidence and cannot always be applied to low-incidence health care settings. To our knowledge, no prospective studies examining the impact of social determinants on TB treatment outcomes have been conducted in high-income, low-incidence settings. Multidrug- or rifampicin-resistant TB (MDR/RR-TB) patients are at increased risk of treatment discontinuation.^4^ A retrospective analysis of two German treatment centres showed 25% inconclusive treatment outcomes due to transfer and loss to follow-up (LTFU) with individualised MDR/RR treatment of 20 months (median).^11^ Shorter and more tolerable regimens like BPaL/M (bedaquiline, pretomanid, and linezolid with or without moxifloxacin) promise fewer side effects and higher rates of treatment completion.^12,13^

Since 2022, the WHO guidelines prioritise the use of the 6-month BPaL/M regimens for treatment of MDR-TB. Despite challenges with the implementation of this pretomanid-containing regimen, the recommendation was adopted for MDR/RR-TB in Germany in 2023.^14^ Other shortened 6- and 9-month treatment regimens based on the results of EndTB^15^ and BEAT-TB^16^ trials were included in the updated WHO guidelines 2025, and further adoption of national guidelines will be necessary.^17^ Recent study data and the evolution of guidelines will lead to a variety of different treatment options for MDR/RR-TB. However, data on the impact of shortened regimens like BPaLM on treatment outcomes and adherence in low-incidence countries are limited^18^ and not yet available for the new 6- and 9-month treatment regimens.

Both, national and international guidelines emphasise the role of comprehensive care and social support in preventing treatment discontinuation.^17,19^ However, health care providers often struggle to meet the complex sociomedical needs of TB patients in everyday care.^20^ Psychosocial support and overcoming language and structural barriers involve substantial effort. Additionally, migration-related factors, such as residence permit issues and legal requirements, may force patients to relocate during treatment. Financial and political reasons may also lead to cross-border relocation, further complicating follow-up.^20,21^ A recent meta-analysis found that migrants have a 4.3 times higher risk of LTFU during TB treatment compared to the non-migrant population in Europe.^22^ Therefore, German guidelines emphasise the need to provide comprehensive care, if possible, in specialised MDR/RR-TB treatment centres providing the necessary treatment structures.^14,19^ Given the increased risk of treatment discontinuation, MDR/RR-TB patients represent a vulnerable group for research and targeted interventions aimed at improving treatment adherence to avoid unsuccessful treatment outcomes.

In July 2024, the study project ‘No1Lost’ was initiated to explore the reasons for TB treatment discontinuation/LTFU with a strong interest in the relevant multifaceted social determinants. No1Lost is a joint project of CAPNETZ STIFTUNG, the Justus Liebig University (JLU), and the German Central Committee against Tuberculosis (DZK), supported by the German Federal Ministry of Health. As part of No1Lost, the MDR/RR-TB cohort study described in this study protocol analyses prospective quantitative data in the vulnerable subgroup of MDR/RR-TB patients. At the same time, qualitative data will be collected by structured interviews with TB patients in Germany both within and outside the MDR/RR-TB cohort to include the patients’ perspectives.

## STUDY OBJECTIVES

The primary aim of this prospective MDR/RR-TB-cohort study is:

1. to describe treatment outcomes and analyse factors associated with treatment discontinuation/LTFU among MDR/RR-TB patients in German TB treatment centres.
  The secondary aims were to describe:
2. the use, outcomes, and safety of the different available MDR/RR-TB treatment regimens in Germany,
3. adherence to German TB guidelines and explore possible barriers in the German health care system,
4. local treatment structures in German treatment centres relevant for the management of MDR/RR-TB.

## METHODS

### Study design

This is a prospective, observational, multi-centre cohort study. Patients will be observed from January 2025 to December 2027 in German TB treatment centres, recruited from a network of at present 72 clinics available for MDR/RR-TB treatment.^23^ After assessment of feasibility, including number of MDR/RR-TB patients in the last 5 years and prerequisites for successful study implementation, 23 TB treatment centres have been recruited for the study to date. Based on prior case notifications, we expect to enrol approximately 150 patients into the prospective cohort.

### Participants

All patients aged 18 years or older starting MDR/RR-TB treatment at the participating TB centres are considered eligible for the prospective cohort, if written informed consent can be provided. Exclusion criteria include concurrent participation in an interventional study as well as restrictions due to which it is not possible for the patient to give informed consent to participate in the study or to understand and comply with the study requirements. In the event of non-participation, the reasons for study exclusion will be documented and reported.

### Sample size

In Germany, 166 cases of MDR/RR-TB were registered in 2022, an increase from 97 cases attributed to the ongoing war in Ukraine.^3^ This number of cases increased to 208 in 2023.^1^ Data for 2024 are not published yet, but numbers are projected to decline below 200 cases. Based on surveillance data, we anticipate an average of approximately 150 MDR/RR-TB cases per year in 2025–2027. The results of a preparatory workshop and online surveys led us to the assumption that 20 clinics treat over 70% of all MDR/RR-TB cases in Germany. The treatment centres with the highest number of MDR/RR-TB cases participate in the study. Based on our estimates, it will be possible to include 150 MDR/RR-TB cases within the study period, considering the non-participating centres.

### Data collection

For the prospective cohort, routine clinical data concerning TB disease and treatment, along with demographic, sociomedical, radiology, microbiology, and laboratory data, will be collected at hospital admission (baseline, T0) and discharge (T1). During outpatient treatment, follow-up data will be collected every 3 months, restricted to TB treatment, radiology, and microbiology data until a treatment outcome is documented (T2). Post-treatment follow-ups occur every 6 months after treatment completion and include data on outcome changes, recurrence, and post-TB lung disease (T3) (Figure).

**Figure.**
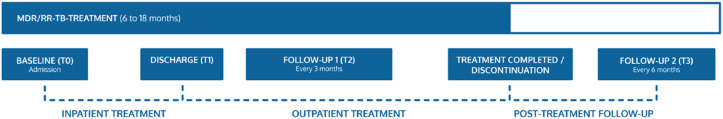
Data collection procedures in the No1Lost MDR/RR-TB-cohort study. MDR/RR-TB = multidrug- and rifampicin-resistant TB; T0–3 = time points 0 to 3.

The study physicians responsible for data collection will be trained through on-site and virtual preparatory meetings to manage a web-based portal using standardised electronic Case Report Forms (eCRFs). The German guideline recommends monthly consultations between outpatient facilities and TB treatment centres,^14,19^ facilitating data collection for the T2 and T3 follow-ups.

### Outcomes and measurements

#### Aim 1

To describe MDR/RR-TB treatment outcomes in German TB centres and to analyse known and discussed factors (covariates) associated with treatment discontinuation. The primary outcome measure for Aim 1 is treatment outcome at the end of MDR/RR-TB treatment, according to the updated WHO definitions.^24^ Successful treatment outcomes include ‘treatment completed’ and ‘cured’. ‘Treatment completed’ applies to patients who finish treatment according to guidelines without falling into the category of unsuccessful treatment, while ‘cured’ requires sustainable microbiological conversion of sputum cultures. Unsuccessful treatment outcomes include ‘died’, ‘treatment failed’, ‘lost to follow-up (LTFU)’, and ‘not evaluated’. Follow-ups after treatment completion are not included in this definition and will not be part of the primary analysis. Nevertheless, during post-treatment follow-ups (T3) relapses will be documented. Treatment interruptions of more than 2 months classify as treatment discontinuation, and if no further information on disease progression or whereabouts is available, patients are classified as LTFU. The category ‘not evaluated’ includes patients where no treatment outcome is assigned due to relocation.^24^ For transfers within the study cohort, data collection will continue until a definitive outcome is documented.

Covariates are used for cohort characterisation and as independent or confounding variables in the statistical analysis. They were selected based on known risk factors for unsuccessful treatment and discontinuation in the literature^5–9^ such as demographic data (e.g., age), health-related factors (e.g., smoking, alcohol use, drug use, comorbidities, and co-infections), and clinical as well as microbiological parameters like the extent of TB disease and sputum smear positivity. Additional covariates represent suspected risk factors, like social determinants and structural barriers, discussed in a preparatory workshop with a diverse group of stakeholders in German public health, clinics, ambulatory care, and social work. These include social determinants such as residency status, socio-economic status, living conditions, and insurance coverage (Table).

**Table.** Study aims, collected outcomes variables, and corresponding time of data collection in the No1Lost MDR/RR-TB cohort study.

Aims	Outcomes variables	Prospective cohort		
		T0	T1	T2, T3
1. To describe MDR/RR-TB treatment outcomes and to analyse known and discussed risk factors (covariates) associated with treatment discontinuation.	MDR-TB treatment outcome^**A**^		X	X
	Treatment completed, cured, died, treatment failed, lost to follow-up, not evaluated			
	Demography	X		
	Age, gender, height, weight			
	Health-related data	X	X	
	Smoking habit, alcohol consumption,^**B**^ drug use, body mass index (BMI), pregnancy, comorbidities (respiratory-, cardiovascular-, liver-, kidney-, psychiatric-, rheumatologic diseases, malignancies, diabetes), co-infections (HIV, hepatitis B and C), solid organ or stem cell transplantation, others			
	Social determinants	X	X	
	Country of birth, migration background, religion and residency status,^**C**^ language/reading skills, socio-economic status^**D**^ (education, occupational qualification, employment, financial resources), living conditions, marital status, social environment at treatment location, insurance status, others			
	TB history and risk	X		
	Previous TB episodes, residence in high-burden country,^**E**^ imprisonment, contact with infectious TB case, BCG vaccination status, use of biologicals, others			
	TB symptoms	X	X	X
	Fever, night sweat, unintentional weight loss, pain, shortness of breath, oxygen saturation, cough enlarged lymph nodes, symptom duration			
	TB drug resistance	X	X	X
	Phenotypic and genotypic resistance testing including sequencing data			
	Infection control		X	
	Isolation measures and duration			
2. To evaluate and compare outcomes and safety of different MDR/RR-TB treatment regimens in German TB centres.	MDR-TB treatment regimens	X	X	X
	BPaL, BPaLM, other 6- to 9-month regimens, individualised treatment			
	Adverse events^**F**^		X	X
	Allergic reactions, skin discolouration, polyneuropathy, impaired vision, nausea, psychological alterations, hepatotoxicity, prolonged QTc time, anaemia and other forms of myelosuppression, renal insufficiency, others			
	Complications		X	X
	TB immune constitution syndrome (TB-IRIS), others			
3. To evaluate guideline adherence.	Covariates and treatment data will be used to decide on performance indicators based on relevance, scientific soundness, and feasibility.	X	X	X
4. To characterise the relevant structures for MDR/RR-TB treatment in Germany.	Treatment structure	X	X	
	Geographical location/region, level of care,^**G**^ number of hospital beds, hospital type,^**G**^ sponsorship,^**G**^ specialised medical departments for MDR/RR-TB treatment, leisure activities/social support for TB patients, language and culture mediation, local network structures, use of assessment tools during treatment (i.e., questionnaires for QoL, psychological stress, and symptoms), others			
5. To characterise the relevant structures for MDR/RR-TB treatment in Germany.	Diagnostic imaging	X	X	X
	Imaging for pulmonary and/or extra-pulmonary TB			
	Laboratory diagnostics	X	X	
	ALT/ALAT, AST/ASAT, GGT, bilirubin, creatinine, haemoglobin, platelets, leukocytes, TSH, QTc time, albumin, interferon-gamma release test, SARS-CoV-2 RNA, streptococcus pneumoniae, legionella pneumophila, others			
	Pathogen diagnostics	X	X	
	Sputum diagnostics (microscopy, PCR, culture), bronchoscopic interventions, CT-guided biopsies, others			

Time points: T0 = baseline; T1 = discharge; T2 = follow-up after discharge; T3 = follow-up after treatment completion.

ALT/ALAT = alanine transaminase; AST/ASAT = aspartate transaminase; BCG = Bacillus Calmette–Guérin; BPaL = bedaquiline, pretomanid, and linezolid; BPaLM = bedaquiline, pretomanid, and moxifloxacin; CT = computer tomography; GGT = gamma-glutamyltransferase; HIV = human immunodeficiency virus; MDR-TB = multidrug-resistant TB; MRI = magnet resonance imaging; PCR = polymerase chain reaction; PET = positron emission tomography; QoL = quality of life; QTc = duration from the onset of the Q wave to the end of the T wave in the heart’s electrical activity; RNA = ribonucleic acid; SARS-CoV-2 = severe acute respiratory syndrome coronavirus type 2; TSH = thyroid-stimulating hormone.

A WHO treatment outcome definition, updated in 2021.^24^

B Alcohol Use Disorders Identification Test (AUDIT-C).^25^

C RKI recommendations for collecting and analysing migration-related determinants in public health research.^26^

D RKI recommendations for calculating index of socio-economic status.^27^

E WHO global list of high-burden countries for TB, HIV-associated TB, and drug-resistant TB.^28^

F The Division of Acquired Immunodeficiency Syndrome (DAVIS) table for grading the severity of adult and paediatric adverse event.^29^

G German Federal Ministry of Health classification for inpatient care facilities.^30^

#### Aim 2

To describe the use, outcomes, and safety of different MDR/RR-TB treatment regimens in German TB centres. Outcomes relevant to Aim 2 include the above mentioned MDR/RR-TB treatment outcome according to WHO definitions^24^ depending on different MDR/RR-TB treatment regimens. Safety will be monitored through systematic documentation of adverse events (e.g., allergic reactions, skin discolouration, polyneuropathy, impaired vision, nausea, psychological alterations, hepatotoxicity, prolonged QTc time, anaemia and other forms of myelosuppression, and renal insufficiency), as well as major complications such as TB-IRIS. Clinical laboratory data and imaging results will support the safety evaluation. The Division of Acquired Immunodeficiency Syndrome (DAVIS) table for grading the severity of adult and paediatric adverse event was used for safety evaluation.

#### Aim 3

To describe adherence to German TB guidelines and explore possible barriers in the participating TB centres. A set of performance indicators will be selected by a panel of guideline authors based on relevance, scientific soundness, and feasibility as described by the Association of the Scientific Medical Societies in Germany (AWMF)^31^ for Aim 3. Staff Interviews based on the results of interim analyses will be used to generate qualitative data to explore reasons and possible barriers for non-adherence to German guidelines.

#### Aim 4

To describe the clinical treatment structures for MDR/RR-TB treatment in the participating TB centres. Covariates relevant to Aim 4, such as hospital size, geographical setting (urban/rural), local network structures, hospital sponsorship, the availability of specialised departments for MDR/RR-TB treatment and facilities for TB treatment and support, infection control measures, and the use of assessment tools, for example, for quality of life, will be used to describe MDR/RR-TB-treatment structures in the participating TB centres. A comprehensive overview of outcomes, covariates, and the corresponding time points of data collection (T0–T3) is provided in Table.

### Data management

CAPNETZ STIFTUNG with its well-established structure for conducting clinical studies in Germany will be responsible for data management with support of the No1Lost study team.^32^ Study data is stored in the Electronic Data Capture (EDC) system, designed for data collection, management, and monitoring in multi-centre clinical trials. It includes a web-based data entry module, plausibility checks, reporting tools, and a query management system that complies with Good Clinical Practice and FDA 21 CFR Part 11 standards. Protocol-based case report forms (CRFs) are programmed into the EDC system with functional pre-testing prior to data collection. Each study site receives virtual training and optional on-site support. Throughout the study, CRF entries are monitored online for completeness and content discrepancies. Interim analyses will ensure efficient data processing and data quality, including missing value analysis, and are conducted at the end of the first and second year of the study. Summary reports on recruitment status, case numbers, and data quality will be shared with study partners and the clinical network to ensure transparency and collaboration between the study team and TB sites.

### Statistical analysis

Main outcomes (defined in aims 1–4 above: 1) MDR/RR-TB treatment outcome, 2) outcomes and safety of different MDR/RR-TB regimens, 3) guideline adherence, and 4) relevant structures for MDR/RR-TB treatment) as well as covariates (demographic, health-related, sociomedical, and clinical parameters) will be analysed using descriptive statistics. Nominal data will be presented with absolute and relative frequencies, continuous data with means and standard deviations (±SD), and ordinal or skewed data with medians, first and third quartiles (Q1 and Q3). The sample size was calculated to ensure adequate precision for estimating the primary outcome of treatment discontinuation. Based on previous data,^11^ we anticipate a discontinuation rate of approximately 25%. To estimate this proportion with a 95% confidence interval (CI) margin of error of 7.5% or less, a sample of at least 129 patients is required. Our expected enrolment of approximately 150 MDR/RR-TB patients is therefore feasible and exceeds this minimum requirement. With this sample size, we will be able to estimate an observed rate of 25% with a 95% CI of 18.1%–31.9% (margin of error 6.9%). This level of precision is considered sufficient for the study’s primary objectives of informing future programme planning and intervention design (powermediation package in R). In an exploratory analysis, MDR/RR-TB treatment outcome will be modelled as a binary dependent variable in generalised linear models (e.g., logistic regression) to quantify the effect of relevant demographic, sociomedical, and clinical factors on treatment discontinuation (yes/no). The WHO categories ‘LTFU’ and ‘not evaluated’ will be combined and classified as treatment discontinuation. Effect sizes, such as odds ratios with 95% CIs, will be calculated and reported. To account for clustering effects at the TB centre level, we will also employ generalised estimating equations. All statistical tests will be two-sided, and robust standard errors will be applied to improve variance estimation. A significance threshold of *P* < 0.05 will be used, with adjustments for multiple testing when necessary.

### Ethical statement

This study was registered in the German Clinical Trials Register (DRKS00036074) and will be conducted in accordance with Good Clinical Practices. The Institutional Review Board (IRB) of the primary coordinating site at Medical School in Hannover (MHH) approved the No1Lost cohort study protocol (11636_BO_S_2024) as well as IRBs at all participating TB centres. Written informed consent will be obtained by site coordinators using documents approved by the IRBs. A certified translation of written information about study procedures, participation, and data safety will be provided in each patient’s individual language. Data collection was harmonised with existing TB-cohorts within German networks to prepare for data comparison and facilitate future cooperation. The results of this study will be disseminated during national and international scientific conferences and published in peer-reviewed journals. Stakeholders and public funders will be informed by regular interim reports.

## DISCUSSION

The No1Lost cohort study is a prospective observational cohort study aiming to explore the reasons for treatment discontinuation among MDR/RR-TB in Germany. Collaborating with existing TB networks and established study infrastructures of the CAPNETZ STIFTUNG provides a robust foundation for conducting this study with a vulnerable TB patient group in a health care setting, where MDR/RR-TB is rare treatment situation.

While most studies exploring risk factors for MDR/RR-TB treatment discontinuation originate from high-incidence countries,^5–10^ the No1Lost cohort study will provide prospective data in the low-incidence setting of German TB care. In addition to clinical and treatment data, comprehensive data on social determinants and treatment structure will be collected at 23 TB treatment centres. Given the importance of psychosocial support during MDR/RR-TB treatment, this study will add important knowledge about the influence of social determinants on MDR/RR-TB treatment outcomes. The detailed quantitative and qualitative analysis of health care structures in German MDR/RR-TB treatment centres will help to describe barriers and facilitators to develop interventions for improved patient care. Additionally, the implementation of the new WHO treatment guidelines for MDR/RR-TB will be accompanied with clinical data collection to evaluate the uptake of the new WHO treatment recommendations in the German health care setting. While patient numbers in this setting will be relatively small, the study benefits from a detailed data collection with multiple data entry points.

Our study has potential limitations to be considered. The implementation of a TB-specific study structure itself may have an influence on patient care in the participating TB treatment centres. The study design may be susceptible to observation bias, referred to as the Hawthorne effect,^33^ since clinicians will be aware of the data collection process. Efforts will be made to minimise this effect by integrating data collection into routine workflow and limiting external monitoring to the necessary extent. Behavioural changes, such as changes in documentation rates, that is, for side effects, use of diagnostic tools, or specific drug combinations, will be monitored and analysed. Site-specific retrospective data will be available to enable comparison of outcomes before and after the start of the prospective data collection. Behavioural changes will be explored through staff interviews to gather additional information about the impact of observational bias on our study results. At the same time, the analysis of quantitative and qualitative data will be used to evaluate the potential benefits of a national TB registry to continuously monitor and improve MDR/RR-TB patient care.

The No1Lost MDR/RR-TB cohort study provides the first prospective data on MDR/RR-TB treatment in Germany to improve the understanding of risk factors for treatment discontinuation/LTFU in a low-incidence setting. In-depth quantitative and qualitative analyses of the social determinants and clinical risk factors as well as treatment structures will inform the development of future health care strategies to address the high rates of treatment discontinuation/LTFU in this vulnerable population.
